# Crude Edible Fig (*Ficus carica*) Leaf Extract Prevents Diethylstilbestrol (DES)-Induced DNA Strand Breaks in Single-Cell Gel Electrophoresis (SCGE)/Comet Assay: Literature Review and Pilot Study

**DOI:** 10.35248/0975-0851.19.11.389

**Published:** 2019-04-01

**Authors:** Alrena V Lightbourn, Ronald D Thomas

**Affiliations:** Basic & Pharmaceutical Sciences Division, College of Pharmacy & Pharmaceutical Sciences, Florida A&M University, Tallahassee, FL, USA

**Keywords:** Diethylstilbestrol, Fig, *Ficus carica*, DNA damage, Strand break, SCGE, COMET assay, MCF10A, Breast cancer

## Abstract

Fig (*Ficus carica*) trees are among the oldest plants on earth. The chemopreventive properties of constituent polyphenols and fiber that implicate figs in having a functional role in averting cancer have not been fully elucidated. We therefore hypothesized that fig leaf extract would inhibit (or attenuate) DES-induced DNA single-strand breakage in MCF10A human breast epithelial cells. To test this hypothesis, MCF10A cells were treated with DES (1, 10, 100 μM), crude fig leaf extract (5, 10, 15 μL), or concomitant doses of DES (100 μM)/fig leaf extract (5, 10, 15 μL). The cells were analyzed for DNA strand breakage using the SCGE/COMET assay with mean olive tail moment as a marker of DNA damage. DES induced DNA strand breaks at all treatment levels compared to DMSO and non-treatment controls. DES at concentrations of 1, 10, and 100 μM produced mean olive tail moments of 1.2082 (177.6%), 1.2702 (186.7%), and 1.1275 (165.7%), respectively, which were statistically significantly (p<0.05) higher than the DMSO control value (0.6803). Exposure to fig leaf extract produced no DNA damage. Rather, a desirable dose-dependent reduction in DES-induced DNA strand breaks was observed. Composite treatment of MCF10A cells with DES and fig leaf extract attenuated DES-induced DNA strand breaks. Taken together, these results suggest a potential mechanism for cancer chemoprevention. Additional studies are necessary to identify relevant active ingredients, confirm the mechanism of action, and further elucidate the therapeutic potential of fig leaf extract for early-stage breast cancer chemoprevention.

## Introduction

Cancer, ranked the second leading cause of mortality in the United States [1] and Florida [2], is further segregated to attribute to breast cancer the designation of No. 1 cause of death among women [3,4]. The most recent global cancer statistics (September 12, 2018) point out an escalating pattern in cancer incidence and mortality based on 36 types of cancer measured in 185 countries of the world. Roughly 18.1 million new cases and 9.6 million cancer deaths are expected globally in 2018 alone. Of these cases, the incidence and mortality of cancer in the Americas (North, South and Central America, and the Caribbean), is estimated at 21.0% (_~_3.8 million) and 14.4% (_~_1.4 million) cases, respectively. The incidence of breast cancer ranks second (11.6%) and deaths fifth (6.6%) among the most commonly diagnosed cancers (lung, breast, colorectal, stomach, liver and prostate) in both sexes. Female victims of cancer represent 24.2% (8.6 million) of new cases and roughly 15.0% (4.2 million) of deaths [5]. Overall, from 2017-2018, *in situ* cases are the primary form (28%) of breast cancer in women between 50-69 years old; invasive cases prevail (27%) between 60-69 years old; and deaths are most prevalent (27%) ≥ 80 years old [1,6,7].

Disparities exist in the incidence of female breast cancer based on data from 1975-2014, which indicate a higher rate of smaller (<2.0 cm) tumors per 100,000 Blacks compared to White females with larger tumors (2.0 – 4.9 cm). A closer review of the overall death toll among Black females with breast cancer from 1975-2015 revealed a more subtle overall decline in mortality than the more prominent reduction in mortality rates noted among White females. In 2015, Black women experienced _~_39% higher death rates (29.5 per 100,000) than Whites (20.8 per 100,000), a difference possibly attributed to differences in associated risk factors (e.g., socioeconomic conditions, comorbidities such as obesity. The 5-year survival rate was a meager 9% following diagnosis [6].

Early intervention is imperative for increasing the chance of recovery from treatment of breast cancer. Ductal or lobular malignancies may initially be detected as a lump or other visible change in the morphology of the breast. General risk factors that increase the chance of developing breast cancer may include: family history of the disease in immediate female family members; personal history of benign breast tumor; personal history of invasive, *in situ* or lobular malignancy; dense mammary tissue; inherited genetic mutations of breast cancer genes (e.g., BRCA1, BRCA2); early onset of menarche; older age of first birth; exposure to radiation; obesity; alcohol consumption; sedentary lifestyle; hormonal treatment of menopause [8], and high fat consumption [9]. Current clinical interventions for breast cancer include screening measures such as: clinical breast exam; screening mammography; breast tomosynthesis; breast ultrasound; breast MRI [10]; thermography; tissue sampling via fine-needle aspiration, nipple aspiration, or ductal lavage; chemotherapy; adjuvant therapy; surgical removal of the breast or relevant area of tissue [8]. An annual mammogram is recommended for women ≥ 40 years old, and certain high-risk (e.g., history of breast cancer in immediate family; genetic predisposition) subgroups of women ≥ 30 years old may be subject to varying combinations of mammography, MRI or ultrasound procedures annually [6]. Due to the invasive nature of some existing screening methods, as well as the increased risk of cancer growth from radiation exposure, alternative approaches are needed both to prevent and treat female breast cancer. Since the 1990s, alternative medicine has helped to bridge gaps in therapy left by modern pharmaceuticals [11,12], which in part, have been unable to cure longterm conditions (e.g., bronchitis, arthritis, rheumatism, heart disease, back pain, high blood pressure, ulcers, etc.) and sometimes chronic degenerative diseases of aging [ 13]. Hitherto, a wide array of studies has demonstrated an inverse correlation between cancer incidence and the intake of fruits and vegetables [8,14].

Edible fig (*Ficus carica L.*) trees are dicotyledonous, perennial plants belonging to the Moraceae (Mulberry) family. Native to Egypt or Western Asia, fig trees were introduced into Middle Eastern and European civilizations, as well as various regions of the United States: New England (MA); Mid-Atlantic (NY, PA); South Atlantic (FL, NC, SC, VA, DC); East South Central (AL, MS, TN); West South Central (LA, TX); and Pacific (CA) [15–19]. The United States, Turkey, Greece and Spain are among the largest producers of fig in the world [15]. In the U.S. consumption of figs is measured both by imports and exports of this commodity. Approximately 27 million pounds of fresh or dried fig valued at roughly $47 million was imported from 2017-2018. National agricultural statistics for the same period showed that 6.9 million pounds of fig were exported for a new gain of almost $14 million [17,18]. Production in the state of California accounts for 98% of U.S.-grown figs [20], which may be incorporated into fig paste, concentrate, powder, or nuggets, or simply diced or sliced [15,21].

Medicinal [22,23], folkloric [24] and biblical [25] uses of figs have been documented for centuries. Figs are fat-free, low in sodium, and cholesterol-free [15]. Figs are a nutrient-rich dietary source of natural sugar, vitamins (A, Bl, B2, B3 and C), minerals (potassium, zinc, magnesium, iron, nitrogen, calcium, phosphorus), fiber and antioxidants (polyphenols) [15,26]. Non-nutrient components found in figs include: benzaldehyde and coumarins (i.e., angelicin, marmesin, psoralen, umbelliferone, and bergapten) [15]. Constituents of fig leaf include: phytosterols such as beta-sitosterol and taraxasterol; and furanocoumarins such as: psoralen and bergapten, [15,23,27]. Latex (a white milk) may be obtained from fig fruit, twigs, as well as from fig leaves. Ficin, a proteolytic enzyme capable of dissolving growths such as corns and warts [22–24], is found in fig leaves.

The biological and ecological importance of the fig tree cannot be overlooked. Wild fig is revered as a “keystone fruit,” meaning that it is essential for the survival of other plants and animals [28]. The fig plant is described as a remedy for at least forty different health conditions and its health benefits are associated with cardiovascular, respiratory, digestive, urinary, integumentary, muscular, immune, hepatic, reproductive and endocrine systems of the human body [27]. Medicinal and biological uses of fig leaves, fruit, roots, or bark include its application as follows: aphrodisiac for sterility, endurance, or erectile dysfunction [29,30], laxative [22], relief for sores and sore throat [22], antibacterial, antiviral, antifungal, anti-diabetes [22,31], antioxidant, anti-cancer, hepatoprotective, hypoglycemic, hypolipidemic, anti-HSV, antipyretic, anti-tuberculosis, nematicidal, anti-spasmodic, anti-platelet, anti-helmintic, and anti-mutagenic activity [22,29,32–34]. Figs have also been found to lower the risk of Alzheimer’s disease [35], treat piles (hemorrhoids), and restore skin and hair health [36]. Fig is a folkloric emmenagogue, which can stimulate menstrual flow in the absence of a regular period (amenorrhea) [24]. In ancient biblical days, it was known as the “forbidden fruit” in the Garden of Eden [37], and later King Solomon is said to have applied fig juice to boils [24]. Because of its broad reach, the fig plant is thought to hold great promise for the future of phytomedicine.

Although the mechanisms of fig action on human health have not been fully elucidated, the ubiquity of polyphenols and its high flavonoid content suggest a strong anticancer potential. These substances, common to citrus fruits, are known for protections afforded through: exertion of antioxidant effects; enhancement of the body’s innate detoxification system via cytochrome P450 (CYP450) monooxygenase system; and regulation of enzymes produced by cancer cells [13,15]. Known health benefits attributed to figs include: weight loss; lowering cholesterol; prevention of constipation, heart disease, colon cancer, hypertension, macular degeneration; diabetes control; throat pain relief; urinary calcium loss; venereal disease; strengthens bones; bronchitis; aphrodisiac for sexual dysfunction.

Issues surrounding the treatment of breast cancer are challenging and controversial. Early detection of breast cancer is made possible through the administration of a low-dose x-ray (mammogram), which for many years was recognized as the only screening tool proven to decrease breast cancer mortality rates [10,6]. The Radiological Society of North America (RSNA) reported on December 1,2009 that exposure to therapeutic, low-dose radiation during annual mammograms, as well as repeated exposures, may actually enhance the risk developing cancer in non-diagnosed individuals who may have a familial or genetic predisposition [10]. Odds ratios among high-risk subjects were 1.5× higher than that observed in similar high-risk females devoid of radiological exposure [10,38,39]. On considering breast screening [40–43], several researchers found that supplementing the mammogram with tomosynthesis dramatically enhances the detection of breast cancer [42,43]. Similarly, RSNA [10] reports that more breast cancers are detected with combined digital measures than with any one alone [44]. Commonly observed comorbidities have also been reported among breast cancer survivors [45]. In other postoperative uses of adjuvant radiotherapy to eradicate residual cancer cells, radiotherapy was found to reduce breast cancer mortality, but rather increase cardiovascular disease and lung cancer in the United States [46] and in Germany [47]. Death from heart disease after longterm radiotherapy for breast cancer was also observed by Bouillon and associates [48]. It is possible that the medicinal properties of figs in almost every system of the human body may attenuate breast cancer as well as other comorbidities.

**Figure 1:**
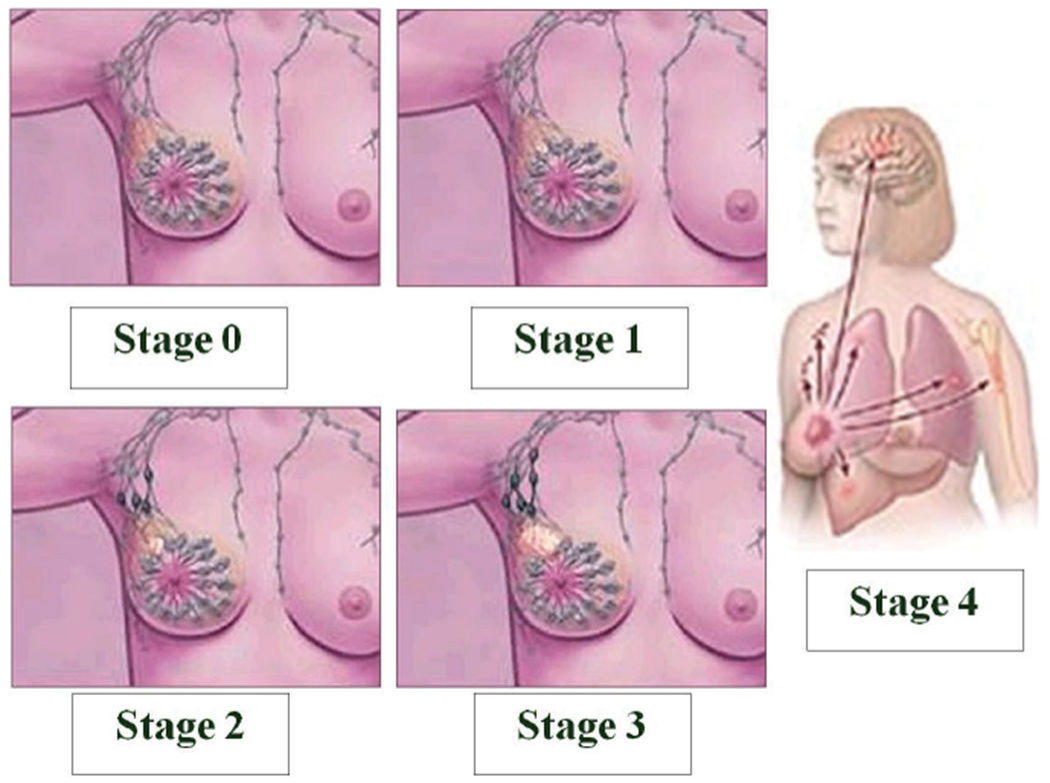
Stages of breast cancer development. Progression of breast cancer in females over four medically recognized stages: Stage 0 (early diagnosis of localized malignancy in breast ducts or milk glands); Stage 1 (cancer dislodges and can invade healthy, intact tissue such as fatty breast tissue, or to a lesser extent, lymph nodes); Stage 2 (onset of cancer growth, spread or both); Stage 3 (cancer is more resistant to treatment but has not contacted bones or organs); Stage 4 (cancer has metastasized from breast and lymph to other parts of body). Figure adapted from http://www.arimedex.com.

To elicit their protective effects, phytochemicals interact with a variety extracellular structural components as well as intracellular molecules, pathways and organelles, thereby counteracting the development of cancer and non-cancer, chronic diseases in the human body. Cancer is a disease commonly characterized by genetic mutation, unregulated cellular proliferation, and aberrant tumor growth and development. The relevance of plant chemicals in cancer prevention is of particular interest to researchers who recognize the importance of identifying specific areas of the multistage process of carcinogenesis where they are most effective. Mammary cancer is also a multi-stage process (Figure 1) that can be induced by chemicals, radiation, viruses, or genetic factors [49]. Absent the timely detoxification and elimination of procarcinogenic chemicals from the body, subsequent absorption and metabolism of cancer-causing agents can lead to the formation of reactive metabolites that may impose more deleterious effects than the parent compound from which they were derived. In recognition of the critical interface between phytochemicals and the process of carcinogenesis, Michael Sporn coined the term ‘chemoprevention’ [50,51] to describe substances capable of inhibiting, reversing or retarding tumorigenesis [14].

The term ‘chemoprevention’ also embodies the two major functional classes of chemopreventive agents: those that either block procarcinogenic insult of normal cells (e.g., ellagic acid, indole-3-carbinol and flavinoids) and those that suppress or retard the transformation of initiated cells into neoplastic lesions (beta-carotene, curcumin, genistein, resveratrol and capsaicin) [14]. However, based on these findings, there remained a gap in the discovery of agents capable of inhibiting, reversing, or retarding that rate-limiting, rapid, irreversible first stage of *carcinogenesis* (i.e., initiation) through which heritable genetic changes may occur. It is in the initiation stage of carcinogenesis that physical interaction of a procarcinogenic, promutagenic substance with DNA leads to DNA damage [52]. Direct action of electrophilic carcinogens can produce highly reactive, nucleophilic metabolites that covalently bind to DNA, causing DNA-adduct formation, and exerting genotoxic effects. Interactions with reactive oxygen, nitrogen or sulfur species may also be genotoxic [52,14]. At the time of this research, the mechanism of natural products derived from plants, and having chemopreventive properties, had not been fully elucidated. Moreover the focus of understanding mechanisms of action of chemopreventive substance such as the *Ficus carica* leaf extract we studied relative to malignant neoplasms versus benign conditions was still in its infancy.

Diethylstilbestrol has generally been classified as a non-*genotoxic (epigenetic)* chemical carcinogen with a hormonal mode of action [52]. Epigenetic carcinogens are sad to exert their effects via mechanisms that “[do not involve] DNA binding, damage, or interaction of the chemical or its metabolites with DNA” [52]. In the wake of these controversies, we hypothesized that fig leaf extract would abrogate or attenuate DES-induced DNA stand breaks. Using the single-cell gel electrophoresis and the comet assay, the objectives of the associated pilot study towards realizing this goal led us to: (1) establish baseline deoxyribonucleic acid (DNA) damage in untreated human breast epithelial cells; (2) determine the effect of fig leaf extract alone on MCF10A cells; (3) assess the integrity of DNA following carcinogen exposure of MCF10-A cells to DES, or its metabolite, DESQ; and (4) assess the ability of fig leaf extract to eradicate DES-induced nuclear effects in MCF10A cells. These preliminary data form the basis of our suggestion that fig leaf extract demonstrates both chemoprotective and chemopreventive properties. Our findings not only warrant reconsideration of DES as a genotoxic agent, but also provide evidence for a phytochemical intervention directly targeting carcinogenesis stage 1 (initiation). The insights from this study fuel the need for more breast cancer research involving this agent, and show promise for the future clinical utility of *Ficus carica* leaf extract for combating early-stage breast cancer development.

## Materials and Methods

### Cells and chemicals

Immortalized, non-transformed, non-tumorigenic (benign) human breast epithelial (MCF10A) cells derived from a 36 year-old, Caucasian female with fibrocystic breast disease were purchased from American Type Cell Culture Collection (ATCC), Rockville, MD). Chemicals used included Dulbecco’s Modified Eagle’s Medium (DMEM), streptomycin, phosphate-buffered solution (PBS), and trypsin, and were purchased with disposable supplies purchased from Sigma Chemical Company (St. Eouis, MO). All refrigerated solutions were brought to room temperature before use.

### Cell culture

Human breast epithelial (MCF10A) cells were sub-cultured twice weekly in serum-free Dulbecco’s Modified Eagle’s Medium (DMEM) supplemented with streptomycin. Incubator settings were 5% CO_2_, 95% air at 37 °C, and 100% humidity. The cells were maintained as exponentially-growing monolayers until confluency was achieved. The cells were washed in PBS, trypsinized, and resuspended in DMEM before treatment.

### Aqueous extraction of the leaves of *Ficus carica*

Fig leaf extract was prepared and refrigerated for future use in research experiments. According to lab protocol, fig leaves were weighed and twice-boiled in water for 30 minutes and the extract vacuum filtered. The extract was treated with 3 ml of 1% HCl per gram of leaf, centrifuged at 3000 rpm for 10 minutes, and the supernatant filtered by vacuum filtration. The extract was concentrated to 50 ml and the pH adjusted to 7.4.

### Analysis of DNA strand breaks by COMET assay

**Figure 2:**
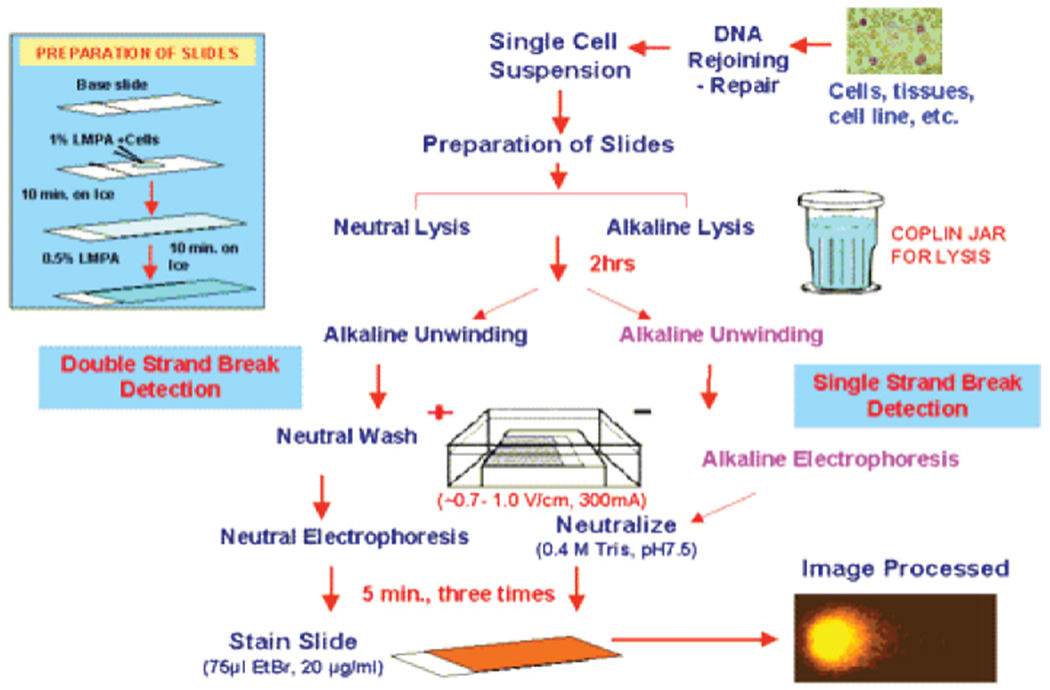
Stepwise COMET assay procedure. The multistep process begins with cell growth and treatment. Suspended cells are fixed to slides prior to alkaline lysis, unwinding and electrophoresis. Neutralized slides can be stored in the refrigerator and away light until imaging is necessary. Slide are individually stained with propidium iodide immediately before fluorescence imaging and comet scoring.

Single-cell gel electrophoresis (SCGE) or the ‘comet a assay’ is a rapid, sensitive, and reliable biochemical technique (Figure 2) for identifying and quantify DNA damage in individual mammalian cells. In the current study, the comet assay was used to detect varying levels of carcinogen-induced DNA fragmentation in normal breast epithelial (MCF10A) cells. The cells were exposed to varying doses of DES dissolved in dimethyl sulfoxide (DMSO), different volumes of fig extract, or a combination of both for 6 hours. Next, MCF10A cells mixed with low-melting point agarose were coated onto frosted slides. Following overnight incubation in an alkaline lysis buffer, the cells underwent electrophoresis in a fresh alkaline rinse solution at 25V/300mA for 30 minutes. The cells were then neutralized and air-dried in preparation for microscopy. Slides were visualized at 20× magnification using propidium iodide as the fluorochrome. The olive tail moment (OTM) was calculated for 40 randomly selected cells from each sample (n=3) using Kinetic® Imaging Komet software.

### Phase contrast microscopy

The growth of cultures of MCF10A cells and comets resulting from chemical treatment were observed by phase-contrast microscopy using a Zeiss fluorescence microscope. DNA stained with propidium iodide was filtered with green-light (excitation ≈546 nm). Where available, photographs of comets were taken to establish DNA damage and/or repair, and to visualize the migration of tail fragments, which form the pattern of a comet during gel electrophoresis.

### Statistical analysis

The quantitative data (Table 1) represent triplicate assays (mean ± SEM) of samples obtained from independent, *in vitro* experiments. Of the 34 parameters measured, olive tail moment (OTM) – the product of percent tail DNA and the distance between the centers of gravity in the head and the tail [53], was selected. A total of 2000 comets from 51 slides were assessed. SAS^®^ software was used to perform statistical analyses of mean olive tail moment, by treatment group. The distribution of the continuous variable (“treatment”) was investigated using the PROC UNIVARIATE procedure. For all treatment groups, there existed a statistically significant difference between treatment means and zero (p<.0001, denoted ‘_***_’). Differences between specific treatment groups determined by one-way analysis of variance (ANOVA) were further assessed using the Tukey, multiple comparisons, post-hoc test. Statistical significance was set at an alpha level of p<.05 (denoted ‘_*_’). Where appropriate and convenient, some graphs were sketched in Microsoft Excel.

## Results

### Sensitivity of COMET assay

**Figure 3:**
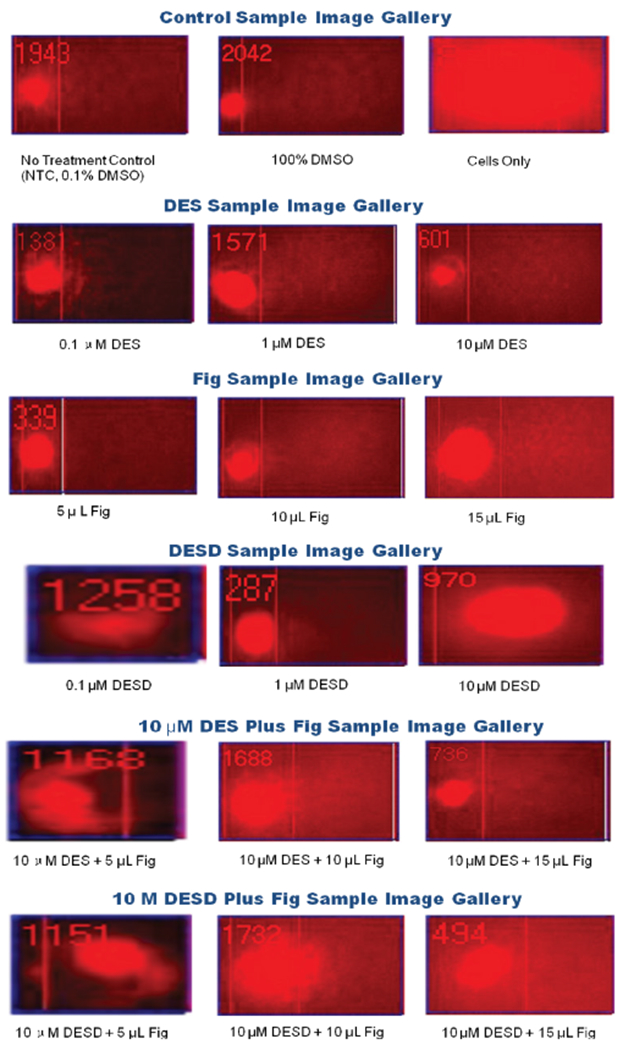
Comet profiles of MCF10A cells in the presence or absence of stimulus. Sample gallery of comets representing DNA strand breaks, fragmentation, and migration of fragments.

The SCGE/COMET assay is a sensitive, non-radiometric procedure. This technique was effective in assessing DES-induced DNA damage in benign human breast epithelial (MCF10A) cells as well as measuring the extent of nuclear insult imposed on MCF10A by environmental estrogens and their metabolites. The protective effects of fig leaf extract in mediating these genotoxic effects were measurable following 6 hours of carcinogen treatment. Microliter quantities of fig leaf extract were also sufficiently potent to evoke cellular changes resulting in DNA repair that was detectable by COMET analysis. Morphological changes in cell structure, particularly intact or disrupted DNA, were visually observed by dark-field microscope (Figure 3).

### Analysis of means

Based on the MEANS procedure in SAS^®^, the null hypothesis that the average olive tail moment (OTM) for 17 different treatment groups is equal to zero was rejected (p<.00005), versus the alternative hypothesis that individual treatment means are not equal to zero. We conclude that the average OTM is different for each type of treatment, indicating that the true mean is greater than zero. This conclusion was also confirmed via the ANOVA procedure in SAS^®^. Data are presented as Mean ± SEM.

#### Baseline DNA damage in untreated MCF10A cells

To measure the occurrence of baseline (spontaneous) DNA stand breaks within human epithelial breast cells, untreated MCF10A cells incubated in growth medium were monitored for comets. The average olive tail moment for comets scored in 120 untreated MCF10A cells was 0.81 ±0.09 (p<.00005). A score above zero in untreated cells confirms that damage (and repair) has spontaneously occurred to DNA within these cells, which is consistent with self-regulated endogenous processes of homeostasis.

#### Nuclear cryoprotection in DMSO-treated MCF10A cells

Dimethyl sulfoxide is routinely added to mammalian cells to preserve and protect proteins from denaturing during freezing. The effect of cryopreservation on DNA integrity within human epithelial breast (MCF10A) cells suspended in DMSO was monitored for comets. The average olive tail moment for comets scored in 120 DMSO-treated MCF10A cells was 0.68 ± 0.06 (p<.00005). This score attests to the added benefit of DMSO, as 16.4% less DNA damage resulted following DMSO treatment compared to untreated controls (0-81 ± 0.09).

#### Effect of DES on MCF10A cells

DES (the parent compound) induced cell death in MCF10A cells, evidenced by the uptake of propidium iodide by cellular nuclei. Moreover, administration of DES at a concentration of 1 μM (1.21 ± 0.25), 10 μM (1.27 ± 1.14), or 100 μM (1.13 ± 0.10) had the greatest impact in OTM (p<.00005) than all other treatment groups. Although the mean OTM increased in a linear fashion at the lower doses of DES, a slightly lower response is seen at the high dose level. These average scores were respectively 148.7%, 156.3%, and 138.7% above untreated control cell levels (0-81 ± 0.09). Compared to DMSO controls (0-68 ± 0.07), the mean OTM was 178%, 187% and 166% higher in respective DES treatment groups. The dose of DES with the lowest amount of DNA damage was DES 100 μM for this treatment group.

#### Effect of DES-Quinone on MCF10A cells

The extent to which DESQ (a metabolite of DES) induced cell death in MCF10A cells was less than that observed for all doses of DES-treated cells. Administration of DESQ at a concentration of 1 μM (0.87 ± 0.09), but not at concentrations of 10 μM (0.59 ± 0.06), or 100 μM (0.62 ±0.05), significantly increased OTM (107.4%) above untreated control values (0-81 ± 0.09). The latter dose levels were less effective (72.3% and 76.0%, respectively) in causing DNA strand breaks. The reduction seen in mean OTM with increasing dose levels of DESQ was not linear, although the DES metabolite was notably less effective in causing strand breaks than the parent compound. These average scores were respectively 128%, 86%, and 91% of DMSO control values (0-68 ± 0.07). The dose of DESQ with the lowest amount of DNA damage was DESQ 10 μM for this treatment group.

#### Effect of fig leaf extract on MCF10A cells

Aqueous fig leaf extract administered alone attenuated DNA damage at all study volumes (5 μL, 10 μL, and 15 μL). Mean OTM values were 0.71 ± 0.09 (86.9%), 0.69 ± 0.06 (84.3%), and 0.79 ± 0.08 (96.7%), respectively when compared to untreated controls (0-81±0.09). Compared to the DMSO-controls (0-68 ± 0.07), dosing with 5 μL, 10 μL, or 15 μL fig leaf extract elicited roughly comparable differences in average OTM (104%, 101% and 116%, respectively) The volume of fig leaf extract producing the lowest amount of DNA damage was Fig 10 μL for this treatment group.

#### Combined effects of DES (Parent Compound) and fig leaf extract on MCF10A cells

As above, DES administered alone (1, 10, or 100 μM) produced OTMs of 1.21, 1.27, and 1.13, respectively. The effect of high-dose DES (100 μM) was offset by administration of fig leaf extract in volumes of 5 μL (1.01 ± 0.10), 10 μL (0.52 ± 0.04), or 15 μL (0.63 ± 0.07), indicating generally tangible reductions in DNA damage and fig leaf extract-mediated protection at higher doses. Tow volume extract only marginally reduced the amount of DNA strand breaks elicited by high dose DES (Table 2).

OTMs resulting from combined treatments were 124.7% (DES 100-Fig5), 63.5% (DES100-Fig10), and 77.6% (DES100-Figl5) of untreated control levels, and 149% (DES100-Fig5), 76% (DES100-Fig10) and 93% (DES100-Figl5) of DMSO control values. With the exception of the DES100-Fig 5 group, the reductions in DNA damage (comet formation, DNA strand breaks) were substantial. Tow dose fig marginally attenuated DNA strand breaks caused by 100 μM DES. However, doubling or tripling the volume of fig extract significantly abrogated DES-induced DNA damage. The combination of DES 100 μM with Fig 10 μL produced the least amount of DNA damage observed by comet assay for this treatment group.

#### Combined effects of DESQ (Metabolite) and fig leaf extract on MCF10A cells

Following induction of DNA damage by pre-exposure to the metabolite of a carcinogenic xenoestrogen, administration of fig leaf extract resulted in a reversal of these effects, below the level of DNA damage observed in untreated controls. Mean OTM following co-administration of DESQ (10 μM) with increasing volumes of fig leaf extract were 0.66 ± 0.06 (5 μL), 0.715 ± 0.07 (10 μL), and 0.59 ± 0.06 (15 μL), respectively. These values were respectively 81.6%, 107.8%, and 82.2% compared to untreated controls (0-81 ± 0.09), versus 98%, 105% and 86% when compared to DMSO controls (0-68 ± 0.07). High volume fig leaf extract (15 μL) was most successful in inhibiting DES-Q (10 μM)-induced DNA strand breakage.

#### Multiple comparisons of OTMs across treatment groups in MCF10A cells

In ANOVA posthoc multiple comparisons of OTM means, Tukey’s studentized range test identified the following groups as being statistically significantly different from each other:

- DES 1 μM was significantly different from: DMSO control; Fig 5; Fig 10; DES100-Fig10; DESQ-10; DESQ-100; DESQ10-Fig5; DESQ10-Fig10; and DESQ10-Figl5 treatment groups (p<.05).
- DES 10 μM was significantly different from: DMSO control; Fig5; Fig10; DES100-Fig10; DES100-Figl5; DESQ-10; DESQ-100; DESQ10-Fig5; DESQ10-Fig10; DESQ10-Figl5 (p<.05).
- DES-100 μM was significantly different from: DES100-Fig10; DESQ-10; DESQ-100; and DESQ10-Figl5 (p<.05).
- DES-100 μM-Fig 5 μL was significantly different from: DES 100-Fig10 (p<.05).

## Discussion

This *in vitro* pilot study was uniquely designed to investigate the utility of *Ficus carica* L. (fig) leaf extract in lowering the risk for human breast cancer. We hypothesized that fig leaf extract would inhibit DES-induced DNA single strand breakage in normal breast epithelial (MCF10A) cells at a time when the anti-mutagenic potential of figs had only been suggested [54] but not exhaustively researched in the open literature. The pilot study affirmed our hypothesis, providing the first such preliminary experimental data that fig leaf extract attenuates DES-induced DNA strand breaks in MCF10A human breast epithelial cells during the initial stage of cancer development.

**Figure 4:**
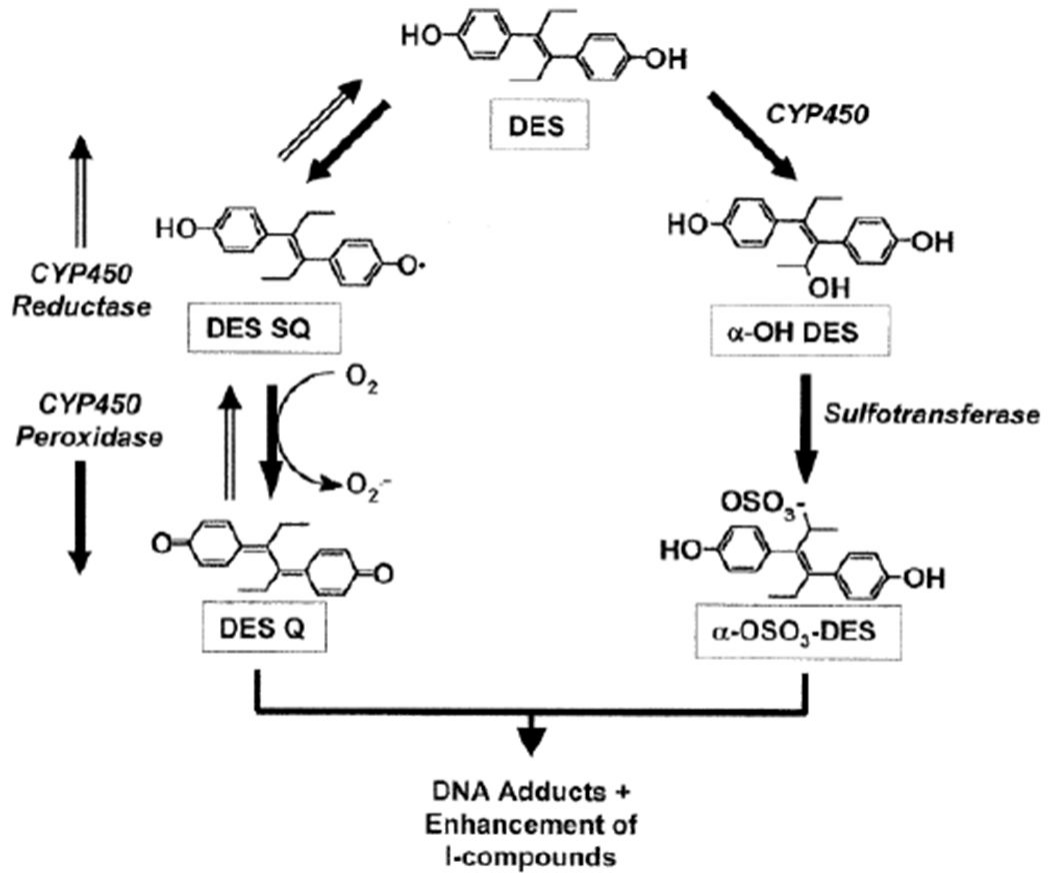
Metabolism of *Diethylstilbestrol.* Conversion of DES to the highly active metabolite, DES-quinone (DES-Q) is a potent initiator of mutations in the DNA structure that cause cancer.

In the multi-step model of carcinogenesis, damage to nuclear deoxyribonucleic acid (DNA) is an essential initiating event for the production of genetic lesions that lead to instability of the genome. Exposure to carcinogenic agents may cause mutations and alter DNA repair and cell cycle control genes. Diethylstilbestrol (DES) is an example of a synthetic estrogenic hormone that is toxicologically characterized as a complete carcinogen. It is known to induce and promote the development of malignant tumors in human breast epithelial cells as well as rodent models. DES is actively biotransformed by cytochrome P450 drug-metabolizing enzymes into its primary metabolically active intermediate, DES-4,4’-quinone (DESQ). The interaction of DESQ with DNA results in the formation of DNA adducts and strand breaks [55]. In the absence of repair, alterations in genes regulating these processes may cause normal cells to be transformed into malignant phenotypes. Replication of these cells can promote the progression of tumorigenesis. The current pilot study showed a stronger potency of the parent compound, DES, than its metabolite, DESQ, where induction of DNA damage was concerned. The destructive effects elicited by the parent compound were consistently more pronounced than those of the metabolite and/or phytochemicals used for chemoprevention. This paradox is interesting because of the three processes commonly implicated in the initiation of cancer in a single cell: metabolism, DNA repair, and cell proliferation [56]. DESQ may be metabolized to either the O- or S- reactive nucleophile (Figure 4) and both are capable to inducing DNA damage [55]. This current finding is also controversial because it intimates that either metabolism is not mandatory for DES to produce genotoxic effects in human breast epithelial cells, or like the traditional view of longterm (≈30 years) development of cancer, the graded effects of low doses of carcinogens over a long time still amount to the same outcome of neoplasm development. Alternatively, it is plausible to consider the presence of a discriminate mechanism whereby DES binds with high affinity to mitogenic ER-alpha, while the action of DESQ is mediated by a ‘deceptively protective’ mechanism at ER-beta [57–61]. Further research is necessary to elucidate these matters.

**Figure 5:**
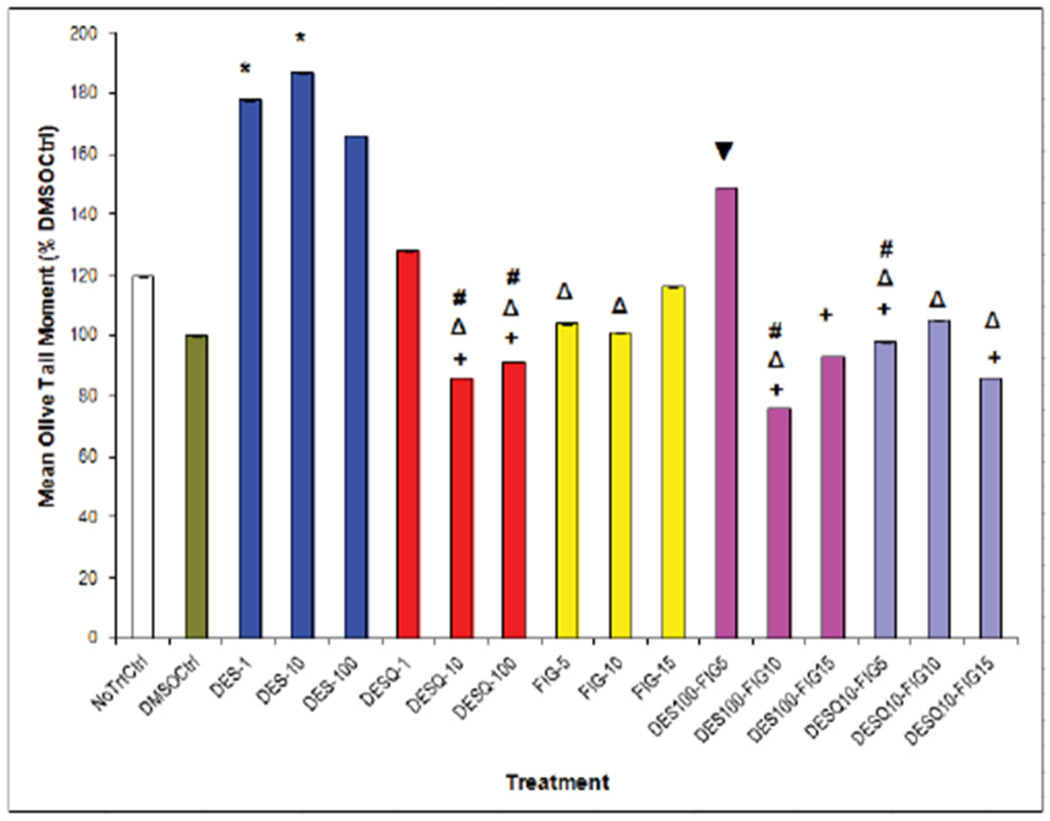
Chemically-induced DNA Damage in Benign Human Breast Epithelial (MCF10A) Cells as Measured by Comet Assay. Effects of DES, fig extract, or combined treatments on MCF10A cells. Induction or attenuation of DNA damage in human breast epithelial (MCF10A) cells with DES (0.1 – 10 μM), fig extract (5 – 15 μL), or high-dose DES plus fig combinations for up to 6 h. NoTrtCtrl = No treatment control; DMSOCtrl = Dimethyl sulfoxide preserved control; DES = Diethylstilbestrol; FIG = *Ficus carica* leaf extract; DES-1, DES-10, DES-100 = DES 1, 10, and 100 μM, respectively; FIG-5, FIG-10, FIG-15 = FIG 5, 10, and 15 μL, respectively. * Compared to DMSO control, p<.05 **+** Compared to DES-10, p<.05 **Δ** Compared to DES-1, p<.05 **#** Compared to DES-100, p<.05 **▼** Compared to DES 100-Fig 10

In this study, low level, DNA damage spontaneously generated within human breast epithelial (MCF10A) cells without exposure to hazardous substances, or with the advantage of exposure to the cryoprotective agent, DMSO. This finding is consistent with limited evidence suggesting endogenous estrogen under certain conditions can itself serve as an initiator of DNA damage [62,63] and increases risk of breast cancer in premenopausal and post-menopausal women [64]. Shifting the microenvironment of cells from estrogen-responsive breast tissue by addition of micoliter quantities of *Ficus carica* leaf extract produced minimal DNA damage relative to experimental controls, implicating its benefits in suppressing tumor cell transformation [65], a potential preference for ER-beta [66], and the well-established observation that a much weaker estrogenic effect is characteristic of phytoestrogens (Figure 5) [66]. These findings also suggest that the fig leaf extract employed in these experiments was roughly as safe as controls, permitting differentiation of its biological activity when used in combination with carcinogens. It is likely that the high phenolic content of fig leaves and the oxygen-scavenging, protective, healing properties of Ficus species [67,68] acts in conjunction with routine innate repair mechanisms of homeostasis within human breast epithelial cells that are benign but capable of activation [63]. In studies by Zhang and associates [69], treatment with *Ficus carica* leaf extract did not compromise the viability of MCF10A cells.

The mechanism of action of DES, its metabolites or analogs is controversial and efforts to elucidate same relative to carcinogenesis continue to emerge. Due to structural similarities, the presence of a phenolic A-ring is critical to receptor binding and estrogenic activity at the estrogen receptor, but not other steroid receptors [9]. Compared to DES, which has two phenol groups at 3-OH and 17-OH, 12.1 Å apart, the distance between these moieties in estradiol is 10.9Å [9]. DES metabolites or analogs that retain these characteristics exhibit significant activity at the ER, but estrogenic activity is abolished without them [9]. DES and estradiol share similar binding affinities (K_a_ of 1.0 × 10^10^ ± 0.8 and 1.5 × 10^10^ ± 0.3, respectively) for the estrogen receptor. DES binds to both ER-alpha and ER-beta [70], while most phytoestrogens exert their anti-estrogenic effects through ER-beta [60,59]. However, whether the carcinogenic potential of the molecule is fueled by the parent compound, or by an oxidative metabolite, has long been unclear [71]. Past research has show DESQ to have tangibly greater activity than DES under certain conditions [72].

In the present study evaluating the carcinogenic properties of DES, it was DES (the parent compound) whose biological activity produced the greater quantity of DNA strand breaks, rather than here weaker DESQ, one of its many reactive oxidative metabolites [72,73]. Here, the necessity for metabolism of this procarcinogen to produce genomic instability was perhaps not the sole determinant of the genotoxic response observed. Both DES and DESQ are able to cross the lipophilic cytoplasmic membrane to enter the cytosol, prior to being translocated into the nucleus for further binding to the estrogen receptor (ER). Rapid metabolism of DESQ by the cytochrome P450 (CYP450) monooxygenase system also provokes a weaker physiological response in affected cells. DESQ binds irreversibly and with only a fraction of the binding affinity of DES, providing a weaker yet persistent stimulus to the ER [74]. DES and DESQ are differentially induce estrogenic and carcinogenic effects, albeit with vastly different rates. The longer stay and persistent low action of DESQ at the ER increases the probability of genomic interactions and may eventually contribute to the promotion of cancer [75]. These findings may also attest to the specific and sensitive nature of single-cell gel electrophoresis (“comet assay”) for detection of superior structural alignment of DES with the estrogen receptor and the resultant genomic disruption [76,77].

On considering the susceptibility of estrogen-sensitive cells to DNA damage, the present study implicates environmental estrogens in triggering the neoplastic process. In our study, MCF10A cells from benign human breast tumor underwent DNA damage without the influence of external stimuli. Addition of DES accelerated the production of DNA strand breaks at all doses. On addition of fig leaf extract to the growth medium, antioxidant constituents of the extract suppressed the DNA damage, thereby preventing the accumulation of DNA strand breaks at all dose levels. In contrast, Zhang and colleagues [69] used *Ficus carica* leaf extracts to suppress neoplastic cell survival, cell cycle and migration in triple-negative breast cancer MDA-MB-231 cells. Besides our reported finings, no other attempt to utilize a *Ficus carica* leaf extract to target the initiation stage of human breast cancer development in non-transformed, human breast epithelial cells was found. Successful *Ficus carica L.* chemoprevention was evidenced by the inhibition and/or reversal of DNA damage (DNA strand breaks) and the apparent promotion of DNA repair following exposure of MCF10A cells to the first synthetic estrogen known to man.

## Conclusion

Diethylstilbestrol, the first synthetic estrogen with non-steroidal carcinogenic potential, has been shown to disrupt the genomic and morphological integrity of non-transformed (benign) human epithelial breast tumor (MCF10A) cells, causing extensive DNA damage (strand breaks and fragmentation) characteristic of the onset of cancer. Cellular metabolism of this carcinogen to its oxidative quinone intermediate (DESQ) was also potent to MCF10A cells.

The prospective contribution of phytoestrogens in alleviating the public health burden of breast cancer is gaining momentum. This pilot study specifically targeted the initiation stage of carcinogenesis, for which natural chemopreventive agents were unavailable in the open literature at the time of this study. From this research, we report that treatment with *Ficus carica* leaf extract inhibits spontaneous DNA damage and reverses non-steroidal estrogen (DES)-induced DNA strand breaks in individual, non-transformed (benign), human epithelial breast tumor cells (MCF10A). *Ficus carica* differentially promotes DNA repair and ameliorates comet formation due to the irreversible interaction of oxidative quinine metabolites of DES (DESQ) with the nuclear apparatus. To our knowledge, this is among the first studies to implicate *Ficus carica* leaf extract in having both a chemopreventive and cancer therapeutic role in early-stage breast cancer.

We, therefore, conclude that *Ficus carica L.* leaf extract is biologically reactive *in vitro* and interacts with the nuclear complex to abrogate DNA strand breaks in MCF10A cells in the presence or absence of diethylstilbestrol (DES) and its oxidative quinine metabolite, 4’,4”-diethylstilbestrol quinine (DESQ).

## Figures and Tables

**Table 1:** Effect of diethylstilbestrol (DES), a xenoestrogen, and its quinone metabolite on human breast epithelial cell (MCF10A) DNA integrity.

Olive Tail Moment (OTM)									
By Slide						By Treatment Group			
Treatment	ID	n	Mean		SEM	N	Mean		SEM
NoTrtCtrl	1a	40	0.863	±	0.169	120	0.813	±	0.090
	1b	40	0.817	±	0.165				
	1c	40	0.759	±	0.132				
DMSO Ctrl	2a	40	0.707	±	0.114	120	0.680	±	0.068
	2b	40	0.767	±	0.109				
	2c	40	0.568	±	0.132				
DES-1 μM	3a	40	2.003	±	0.667	120	1.208	±	0.246
	3b	40	0.754	±	0.257				
	3c	40	0.868	±	0.144				
DES-10 μM	4a	40	1.007	±	0.249	120	1.270	±	0.142
	4b	40	2.089	±	0.285				
	4c	40	0.715	±	0.117				
DES-100 μM	5a	40	0.822	±	0.144	120	1.128	±	0.105
	5b	40	0.984	±	0.121				
	5c	40	1.576	±	0.240				
DESQ-1 μM	6a	40	0.574	±	0.111	120	0.873	±	0.089
	6b	40	0.559	±	0.122				
	6c	40	1.486	±	0.177				
DESQ-10 μM	7a	40	0.754	±	0.142	120	0.588	±	0.059
	7b	40	0.459	±	0.065				
	7c	40	0.550	±	0.079				
DESQ-100 μM	8a	40	0.678	±	0.081	120	0.618	±	0.055
	8b	40	0.649	±	0.128				
	8c	40	0.527	±	0.066				

**Table 2:** Effect of fig (*Ficus carica*) leaf extract on diethystiblestrol (DES)-induced in human breast epithelial cell (MCF10A) DNA integrity.

Olive Tail Moment (OTM)									
By Slide						By Treatment Group			
Treatment	ID	n	Mean		SEM	N	Mean		SEM
FIG-5 μL	9a	40	0.949	±	0.256	120	0.707	±	0.093
	9b	40	0.547	±	0.074				
	9c	40	0.624	±	0.080				
FIG-10 μL	10a	40	0.739	±	0.117	120	0.685	±	0.059
	10b	40	0.720	±	0.098				
	10c	40	0.597	±	0.094				
FIG-15 μL	11a	40	0.965	±	0.160	120	0.786	±	0.082
	11b	40	0.751	±	0.156				
	11c	40	0.643	±	0.099				
DES100+FIG5	12a	40	1.013	±	0.244	120	1.013	±	0.104
	12b	40	1.233	±	0.171				
	12c	40	0.793	±	0.093				
DES100+FIG10	13a	40	0.487	±	0.066	120	0.516	±	0.042
	13b	40	0.561	±	0.089				
	13c	40	0.502	±	0.060				
DES100+FIG15	14a	40	0.537	±	0.075	80	0.631	±	0.066
	14b	40	0.802	±	0.119				
DESQ10+FIG5	15a	40	0.554	±	0.075	120	0.663	±	0.055
	15b	40	0.594	±	0.094				
	15c	40	0.842	±	0.111				
DESQ10+FIG10	16a	40	0.807	±	0.127	120	0.715	±	0.067
	16b	40	0.833	±	0.130				
	16c	40	0.505	±	0.079				
DESQ10+FIG15	17c	40	0.495	±	0.096	120	0.587	±	0.058
	17a	40	0.686	±	0.092				
	17b	40	0.581	±	0.111				

## Abbreviations:

SCGE: Single-Cell Gel Electrophoresis
DES: Diethylstilbestrol
DMSO: Dimethylsulfoxide
DES-Q: DES-4,4’-Quinone
